# Supplementary material for: Chromothripsis during telomere crisis is independent of NHEJ, and consistent with a replicative origin
Source: Genome Res. 2019 May;29(5):737–49. doi: 10.1101/gr.240705.118 (PMC6499312; doi:10.1101/gr.240705.118)
Supplement: Supplemental Material [file supp_gr.240705.118_Supplemental_file_1.zip › contigs/annotated_contigs/DB47/contig.2.DB47_length_640_mean_cov_7.021875.docx]

**DB47_length_640_mean_cov_7.021875**

```
TAGCCTGATTATGCAATAGAAAAGAAAAACCCATTTTCTAGGAAGAAATTCAACCCAGCTGCAGGAATTCTCAGAATTTGCATAAATAA
 >chr14:27758375-27758672 - E=6e-167
CAAGGAGGCTAATGTTAATCACCAAGACAATGGAGAAAATGTACCCAGGGCATGTCAGAGACCTTCTCAGCAGTCCCTTCCATCACAGG

TCCAGAAGCCTAGGAGCTAAATATGATTTCTTGGGCCAGGCCCTGGGTCCTCTTGCTGTGTGCAGCCTAGGGACTTGGTACCCTGTGTC

CCAGTCATCCCAGTCGCTCCAGCCA|TGGCT|GTTTCTCCACCCAAATTTCATCTTGAATTGTAGCTCCCATAATCCCCATGTGTCATA
                          >chr14:27757171-27757519 - E=1e-197
TGAGGGACCTGGTGGGAAGTGATTGGATCATTGGGGGTGGTTTCCCCCATGCTGTTCTTGTGAAAGTGAATAAGTCTCAAGAGCTTTGA

TGATTTTATAAAGGGGAGTTCCCCTGCACACTTTCTCTTGCCTGCCACCATTAAGACATGACTTTTCTCTTCATTTGCCTTCTGCCATG

ATTGTGTGGCCTCCCTAGTCATGTGGAACTGTGTGTCAATTAAACCTCTCTTCTTTATAAATTACCCAGTCTCAGGTATACCTTTACTG

GCAGTGGGAGAATAGACTA
```
